# Targeting Nrf2 Signaling Pathway in Cancer Prevention and Treatment: The Role of Cannabis Compounds

**DOI:** 10.3390/antiox12122052

**Published:** 2023-11-28

**Authors:** Anna Rybarczyk, Aleksandra Majchrzak-Celińska, Violetta Krajka-Kuźniak

**Affiliations:** Department of Pharmaceutical Biochemistry, Poznan University of Medical Sciences, Rokietnicka 3, 60-806 Poznań, Poland; anna.rybarczyk@student.ump.edu.pl (A.R.); majchrzakcelinska@ump.edu.pl (A.M.-C.)

**Keywords:** nuclear factor erythroid 2-related factor 2 (Nrf2), cannabinoids, cannabidiol (CBD), cancer, inflammation, oxidative stress

## Abstract

The development and progression of cancer are associated with the dysregulation of multiple pathways involved in cell proliferation and survival, as well as dysfunction in redox balance, immune response, and inflammation. The master antioxidant pathway, known as the nuclear factor erythroid 2-related factor 2 (Nrf2) pathway, regulates the cellular defense against oxidative stress and inflammation, making it a promising cancer prevention and treatment target. Cannabinoids have demonstrated anti-tumor and anti-inflammatory properties, affecting signaling pathways, including Nrf2. Increased oxidative stress following exposure to anti-cancer therapy prompts cancer cells to activate antioxidant mechanisms. This indicates the dual effect of Nrf2 in cancer cells—influencing proliferation and apoptotic processes and protecting against the toxicity of anti-cancer therapy. Therefore, understanding the complex role of cannabinoids in modulating Nrf2 might shed light on its potential implementation as an anti-cancer support. In this review, we aim to highlight the impact of cannabinoids on Nrf2-related factors, with a focus on cancer prevention and treatment. Additionally, we have presented the results of several research studies that combined cannabidiol (CBD) with other compounds targeting Nrf2. Further studies should be directed toward exploring the anti-inflammatory effects of cannabinoids in the context of cancer prevention and therapy.

## 1. Introduction

The nuclear factor erythroid 2-related factor 2 (Nrf2) pathway plays a critical role in cellular defense against oxidative stress and inflammation, making it an attractive target for therapeutic interventions. The Nrf2 pathway regulates the expression of various genes involved in antioxidant responses and detoxification processes, promoting cellular resilience and reducing damage caused by reactive oxygen species (ROS). Dysregulation of this pathway has been implicated in the development and progression of various types of cancer [1]. Cannabinoids, which are naturally occurring compounds derived from the *Cannabis* plant, have shown the ability to modulate the Nrf2 pathway, offering potential benefits in the context of cancer prevention and therapy. By targeting Nrf2, cannabinoids have the potential to restore the balance of oxidative stress and enhance the body’s natural defense mechanisms against tumor growth. Numerous studies have demonstrated the ability of cannabinoids to activate the Nrf2 pathway, leading to increased antioxidant capacity and reduced inflammation within cells. This activation has been associated with inhibiting cancer cell proliferation, apoptosis induction, and suppression of tumor angiogenesis. Furthermore, cannabinoids have been shown to sensitize cancer cells to conventional therapies, such as chemotherapy and radiation, enhancing their effectiveness and reducing side effects. In addition to their direct effects on cancer cells, cannabinoids exhibit immunomodulatory properties, influencing immune cells’ activity in tumor surveillance and elimination. By modulating the immune response, cannabinoids can enhance the body’s ability to recognize and destroy cancer cells, further contributing to their therapeutic potential in cancer prevention and treatment [2]. This review presents the significance of the Nrf2 signaling pathway as a target of cannabinoids or their combinations with other compounds or drugs.

## 2. Nrf2 Signaling Pathways as a Target

Nrf2 is a transcription factor that plays a crucial role in cellular defense against oxidative stress. It regulates the expression of various antioxidant and detoxification genes, helping to maintain cellular homeostasis [3]. The Nrf2 signaling pathway can be activated through canonical and non-canonical mechanisms [4].

Electrophilic compounds and oxidative stress carry out the canonical activation of Nrf2, which involves several steps. First, the disruption of the Nrf2-Keap1 (Kelch-like ECH-associated protein 1) complex occurs due to modifications in reactive cysteine residues on Keap1, such as oxidation or covalent modification by electrophiles. This disruption prevents Nrf2 degradation and allows its accumulation. Second, Nrf2, facilitated by nuclear import proteins, translocates into the nucleus. Once inside the nucleus, Nrf2 forms heterodimers with small musculoaponeurotic fibrosarcoma (Maf) proteins, another family of transcription factors. This heterodimerization further enhances Nrf2 binding to the antioxidant response element (ARE) and promotes gene transcription. Third, the Nrf2-Maf complex recruits coactivators and interacts with the basal transcriptional machinery, initiating the transcription of a battery of cytoprotective genes, including heme oxygenase-1 *(HMOX-1),* NAD(P)H quinone oxidoreductase 1 *(NQO1),* and glutamate-cysteine ligase *(GCL)* [5]. These genes encode proteins involved in cellular detoxification, anti-oxidation, and redox homeostasis. The canonical activation of the Nrf2 pathway is tightly regulated to maintain cellular balance. One of the negative regulators of the Nrf2 pathway is BACH1 (BTB domain and CNC homolog 1), which competes for binding to the promoters of Nrf2 target genes such as *HMOX-1* and *p62* [6]. Aberrant activation or inhibition of Nrf2 can have significant implications for human health. While Nrf2 activation protects against oxidative stress and inflammation, excessive or prolonged activation may contribute to pathological conditions, such as cancer progression. Understanding the intricacies of the canonical activation of the Nrf2 pathway provides insights into the cellular mechanisms that govern antioxidant defense and stress response.

In non-canonical activation, p62 is a crucial mediator connecting the Nrf2 pathway with autophagy and proteasomal degradation. Under normal conditions, p62 interacts with Keap1, promoting the degradation of both p62 and Nrf2 through the proteasome. However, during cellular stress, such as oxidative stress or the accumulation of protein aggregates, p62 undergoes post-translational modifications and forms aggregates. These p62 aggregates can sequester Keap1 and prevent its interaction with Nrf2, leading to the stabilization and nuclear translocation of Nrf2, independent of the canonical pathway. As a result, Nrf2 activates the transcription of target genes involved in antioxidant defense, proteasome function, and autophagy, further enhancing the cellular stress response [7]. Moreover, p62 also serves as a bridge between autophagy and Nrf2 activation. The p62 aggregates can be recognized by autophagic machinery, facilitating their engulfment into autophagosomes and subsequent degradation in lysosomes. This process, known as selective autophagy or aggrephagy, removes protein aggregates and releases Nrf2 from p62-mediated sequestration, activating it. In addition to its role in autophagy, p62 can directly interact with Nrf2 and modulate its activity. The binding of p62 to Nrf2 promotes its stability and nuclear translocation, enhancing Nrf2-mediated gene expression [8]. The non-canonical activation of the Nrf2 pathway through p62 highlights the intricate interplay between cellular stress response, autophagy, and proteostasis. This mechanism ensures that cells can efficiently cope with oxidative stress and maintain protein homeostasis under challenging conditions. Understanding the non-canonical activation of the Nrf2–p62 axis provides valuable insights into cellular adaptive responses and offers potential targets for therapeutic interventions to combat oxidative stress-related disorders and proteotoxicity [9].

## 3. Structures and Mechanism of Action of Cannabinoids 

**Figure 1:**
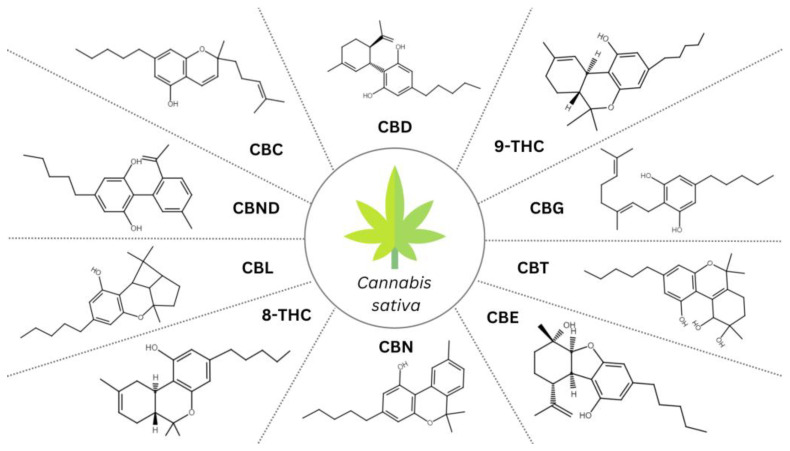
An overview of the structures of representative compounds within phytocannabinoid subclasses; CBD, cannabidiol; 9-THC, (-)-Δ9-trans-tetrahydrocannabinol; CBG, cannabigerol; CBT, cannabitriol; CBE, cannabielsoin; CBN, cannabinol; 8-THC, (-)-Δ8-trans-tetrahydrocannabinol; CBL, cannabicyclol; CBND, cannabinodiol; CBC, cannabichromene.

Cannabinoids constitute a diverse group of lipophilic compounds displaying a terpenophenolic structure with potential biological activity. Endocannabinoids occur naturally within the human body, while a wide variety of exogenous cannabinoids exist, encompassing natural *Cannabis*-produced phytocannabinoids, and synthetic cannabinoids. Phytocannabinoids can be described as natural secondary metabolite constituents of *Cannabis sativa* plants, chemically related to the terpenes with their ring structure derived from a geranyl pyrophosphate [10]. Based on the recent literature, the *Cannabis* plant has been found to contain over 150 phytocannabinoids [11]. They exhibit specific chemical structures (Figure 1), upon which they are classified into 11 different subclasses so far: (-)-Δ9-trans-tetrahydrocannabinol (Δ9-THC), cannabidiol (CBD), cannabigerol (CBG), cannabichromene (CBC), cannabinol (CBN), cannabitriol (CBT), cannabinodiol (CBND), (-)-Δ8-trans-tetrahydrocannabinol (Δ8-THC), cannabicyclol (CBL), cannabielsoin (CBE), and miscellaneous types [10]. It is considered that only CBG, CBD, Δ9-THC, and CBC are originally biosynthesized in *Cannabis*, and in all likelihood, other metabolites are generated through their decomposition [12]. The glandular trichomes on the female flowers are cannabinoid-rich, while the organs devoid of trichomes (roots, seeds) exhibit no presence of cannabinoids; still, the contents of *Cannabis* chemical compositions vary with breeding conditions or the techniques used for extract preparation [13]. Diverse Δ9-THC and CBD ratios in *Cannabis* have led to the identification of three different phenotypes: with a higher content of Δ9-THC (drug-like phenotype I); with the enhanced amount of CBD and concomitant Δ9-THC (intermediate-phenotype II); with a dominant quantity of CBD (fibre-type or hemp phenotype III) [10]. Also, ongoing efforts are being made to improve cultivation methods, including genome editing, to elevate the efficiency of medical *Cannabis* [14].

Over the centuries, interest in *Cannabis* preparations has remained strong due to unraveling the molecular and cellular mechanisms of action predisposing them for medicinal use. Moreover, recreational usage of *Cannabis* has also played a role in the development of synthetic or semi-synthetic equivalents of phytocannabinoids (e.g., dronabinol, nabilone hexahydrocannabinol) or entirely new man-made substances with cannabimimetic effects, usually with enhanced psychoactive properties [15,16]. The phytocannabinoids being the most comprehensively studied in terms of their therapeutic properties are CBD, Δ9-THC, and CBG. A diverse range of activities of cannabinoids has already been described, with particular attention directed towards the management of cancer, neurological and psychiatric disorders, bacterial infections, and dermatological conditions [17,18,19,20]. As an explanation of the psychoactive properties, the interaction with G-protein-coupled receptors—a part of the endocannabinoid system (ECS) called cannabinoid receptors: CB1R and CB2R—is recognized. The central nervous system constitutes the primary location of CB1R, while CB2R is abundantly present within the immune cells [21]. Thus, mimicking endocannabinoids, psychotropic cannabinoids show significant binding affinities to CB1R [22]. In turn, cannabinoids devoid of intoxicating effects—CBD, CBG, CBC, and cannabivarin (CBV)—are weak agonists or antagonists for cannabinoid receptors [23,24]. CBD antagonism of the CB1R constitutes one of the proposed mechanisms, reversing the effects of Δ9-THC, which provides the ability to attenuate psychotic-like symptoms [25]. Nonetheless, it has been suggested that an additional underlying mechanism of the antipsychotic effect arises from an increase in serum anandamide levels, activation of 5-HT1A receptors, transient receptor potential vanilloid type 1 (TRPV1), G protein-coupled receptor 55 (GPR55), and potentially various other mechanisms [26,27]. A growing body of evidence suggests that the neuroprotective involvement displayed by CBD may be associated with peroxisome proliferator-activated receptor-γ (PPARγ) activation [28,29]. Also, CBD can mitigate inflammation due to adenosine release caused by A2A adenosine receptor (A2AR) activation and equilibrative nucleoside transporter inhibition [30,31]. The modest agonistic affinity of CBD at the human serotonin receptor 5-HT1A results in anxiolytic effects observed after administration into the intra-dorsal periaqueductal gray and intra-prelimbic prefrontal cortex at low-moderate doses in a *Cannabis* model [32,33]. Furthermore, the indirect activation of somatodendritic 5-HT1A autoreceptors in the dorsal raphe nucleus mediates the alleviation of nausea and vomiting [34].

*Cannabis* compounds, both alone (particularly CBD) and in complex extracts, and their combinations with drugs have demonstrated cytotoxic effects regarding various cancer types. The literature has demonstrated that the superfamily of transient receptor potential (TRP) channels is an essential molecular target of phytocannabinoids in terms of their anti-cancer properties. CBD and Δ9-THC, CBG, CBC, or CBDV interact with several representatives of these ion channels, mostly acting as agonists and causing an intensified Ca^2+^ influx [24]. The activation of the TRPV2 channel triggered by CBD has been reported to be beneficial regarding anti-cancer outcomes due to the induction of autophagy and/or apoptosis processes and enhancing the standard therapy effectiveness in human endometrial cancer [35], breast cancer [36], leukemia [37], and glioblastoma [38]. The high level of free radicals is usually observed in cancers; however their overproduction can be beneficial, leading to stress-induced tumor cell death [39] An upset in intracellular Ca^2+^ balance prompts the uptake of these ions into mitochondria, hypothesizing it as a potential origin for the production of ROS [40]. Additionally, treatment of colorectal cancer cells with CBD has been shown to elevate mitochondrial ROS levels, supporting the proposed thesis [41]. CBD-induced TRPV4 activation triggers the ER stress response in glioma cells, leading to lethal mitophagy via the ATF4–DDIT3–TRIB3–AKT–mTOR axis [42]. The inhibition mechanism involving tetrahydrocannabivarin (THCV) for the oncochannel TRPV6, which is overexpressed in malignancies, has been suggested by recent research conducted by Neuberger et al. [43]. However, in a murine in vivo model, CBD, by exerting its inhibitory effects on the GPR55 receptor, demonstrated a significant reduction in the proliferation of pancreatic cancer cells [44]. Overexpression of apoptosis-related proteins p53, Bax, and caspase-3, suppression of X-linked inhibitor apoptosis (XIAP), and ER stress activation via Noxa and ROS are also the mechanisms underlying the augmented death of cancer cells treated with CBD [45,46,47]. By upregulating the expression of death receptor 5 (DR5), CBD, in combination with TNF-related apoptosis-inducing ligand (TRAIL), induced tumor size reduction and intensified apoptosis in colorectal cancer [48]. A pharmacogenomics study pointed out that in GBM, CBD tumor suppression properties proceeded from preventing NF-κB subunit RELA phosphorylation on serine-311 and promoting RELA DNA binding [21]. Hence, the mechanisms of *Cannabis* anti-cancer properties are still being elucidated, and novel research may propose alternative explanations.

## 4. Cannabinoids and Regulation of the Redox Balance

**Figure 2:**
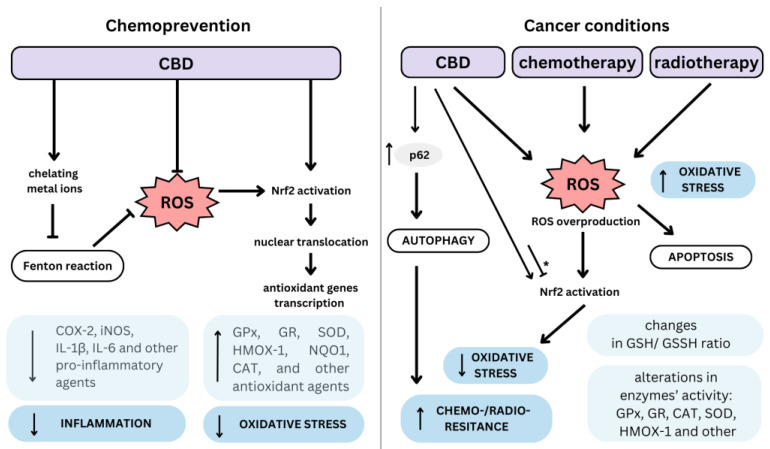
Dualistic mechanism of cannabidiol (CBD) as a chemopreventive and anti-cancer agent. An arrow-ended line indicates cellular stimulation, while a dash-ended line represents cellular inhibition. The asterisk (*) denotes variability in the impact of the stimulus or inhibition depending on the type of cells involved; ROS, reactive oxygen species; COX-2, cyclooxygenase-2; iNOS, inducible nitric oxide synthase; IL-1β, interleukin-1β; IL-6, interleukin-6; GPx, glutathione peroxidase; GR, glutathione reductase; SOD, superoxide dismutase; HMOX-1, heme oxygenase-1; NQO1, NAD(P)H dehydrogenase quinone 1; CAT, catalase; GSH/GSSH, reduced/oxidized glutathione.

Cannabinoids present a multi-directional redox modulatory activity with both antioxidant and pro-oxidant effects [49]. Such pro- and antioxidant functions of cannabinoids may be cell and model dependent and may also be influenced by cannabinoid dose, treatment duration, and underlying pathology [50]. Figure 2 provides an overview of CBD’s divergent effects, depending on cell status.

The antioxidant properties of 9Δ-THC, CBD, several synthetic cannabinoids, and *Cannabis sativa* extracts have been reported in numerous studies, including cyclic voltammetry, in vitro, and in vivo studies [51,52]. In this regard, both 9Δ-THC and CBD exhibit antioxidant activity comparable to that of vitamins E and C [53,54]. However, a higher antioxidant potency for 9Δ-THC than for CBD has been reported [51,52].

As presented above, the antioxidant properties of phytocannabinoids rely on their impact on the levels of the two master regulators of oxidative stress responses, namely Nrf2 and BACH1 transcription factors [6]. Nrf2 is a redox-sensitive transcription factor [55]; thus, the direct influence of cannabinoids on the redox balance indirectly influences the expression of Nrf2. In this regard, the regulation of the cellular redox balance is maintained by cannabinoids via several mechanisms. It is suggested that the phenolic groups readily oxidized to quinoid forms and unsaturated bonds found in non-olivetolic fragments of 9Δ-THC and CBD could be responsible for their antioxidant properties [53]. Moreover, CBD, like other phenolic antioxidants, interrupts free-radical chain reactions and reduces the production of ROS by chelating transition metal ions involved in the Fenton reaction [56]. Regulation of the redox balance by *Cannabis* compounds is also maintained by the indirect (through regulating the expression of antioxidant enzymes) interaction with the components of the redox system [6]. In this regard, CBD increases the activity of glutathione peroxidase (GPx) and reductase (GR) and, in human cardiomyocytes, was found to increase the mRNA level of superoxide dismutase (SOD) [57]. Another mechanism by which CBD exerts its antioxidative effects is by ameliorating dysfunctional mitochondria, a major endogenous source of ROS [58]. Liu et al. showed that CBD could reduce caspase-1/interleukin-1β-mediated mitochondrial ROS generation in H_2_O_2_-treated human keratinocytes by binding to caspase-1 directly [59]. Moreover, CBD could relax the muscle cells of the pulmonary artery by normalizing the mitochondrial morphology and repairing mitochondrial energy metabolism under hypoxic conditions in murine models of pulmonary arterial hypertension [55]. In another study, Dos-Santos-Pereira et al. used mouse microglial cells in culture activated by lipopolysaccharide (LPS) to study the anti-inflammatory potential of CBD [60]. They showed that CBD prevents LPS-induced microglial inflammation by inhibiting ROS/NF-κB-dependent signaling and glucose consumption [60].

CBD also reduces oxidative metabolism in polymorphonuclear leukocytes [61] and H_2_O_2_-treated nucleus pulposus cells [62]. In the latter study, pre-treatment with CBD suppressed the promotion of COX-2, iNOS, IL-1β, and IL-6 expression in the nucleus pulposus cells following H_2_O_2_ exposure [62]. CBD and 9Δ-THC reduced the oxidative stress parameters in aged pancreatic cells [63]. Furthermore, tests in rats indicated that 9Δ-THC and CBD prevent hydroperoxide-induced oxidative damage to neurons [64]. The neuroprotection observed with CBD and 9Δ-THC was unaffected by cannabinoid receptor antagonists, indicating it to be cannabinoid receptor-independent [64]. Interestingly, CBD was more protective against glutamate neurotoxicity in this study than ascorbate or alpha-tocopherol [64]. In another study, CBD attenuated neural production of ROS following cadmium chloride treatment in a manner similar to vitamin E (α-tocopheryl acetate) [65]. The authors concluded that CBD protects dopaminergic neuronal cells from cadmium [65]. In turn, the neuroprotective role of CBG was evaluated in the LPS-stimulated RAW 264.7 macrophage model by Gugliandolo et al. [66]. CBG pre-treatment reduced the levels of pro-inflammatory markers IL-1β, TNF-α, IFN-γ, and PPARγ and improved the Nrf2 cell antioxidant defense via restoring Nrf2 as well as reducing SOD1 and iNOS protein expression, reversing the effects of LPS [66].

In a study by Musetti et al., the antioxidant properties of the *Cannabis sativa* extracts, as well as pure cannabinoids, were measured in a Cu^2+^-induced LDL oxidation assay [51]. The isolated cannabinoids were found to be less effective in preventing the oxidation of LDL, suggesting a synergistic effect between the diverse phytochemicals found in medicinal *Cannabis* extracts [51]. In another study, cannabinoid oils obtained from plant extracts were characterized by more significant antioxidant activity than those prepared from pure cannabinoids [67]. However, it is important to note that, as far as *Cannabis* extracts are concerned, the selection of extractant and extraction conditions significantly influence the active compounds’ extraction efficiency and thus antioxidant activity [68]. In a recent study, the antioxidant potential of the *Cannabis* extracts from Białobrzeskie, Tygra, and Henola hemp cultivars, obtained by ultrasound-assisted extraction and maceration by methanol, ethanol, isopropanol, and their 50:50 (*v*/*v*) mixtures, was studied [68]. All of the extracts have been found to possess antioxidant properties; however, Białobrzeskie leaf extract obtained with ultrasound-assisted extraction with methanol was determined to be the most potent antioxidant [68].

## 5. Cannabinoids as Modulators of Nrf2 Pathway—The Role in Chemoprevention and Cancer Therapy

*Cannabis* and its active compounds, including CBD, have garnered significant attention due to their potential health benefits. One intriguing aspect of their effects is their interaction with the Nrf2 pathway (Table 1). Understanding how *Cannabis* and CBD can modulate the Nrf2 pathway provides insights into their potential therapeutic applications. Using a model of LPS-activated microglia cells, Juknat et al. observed that CBD, and less so ∆9-THC, induce a cellular stress response via modulation of the Nrf2/HMOX-1 axis and the Nrf2/ATF4-Trib3 pathway [69].

**Figure 3:**
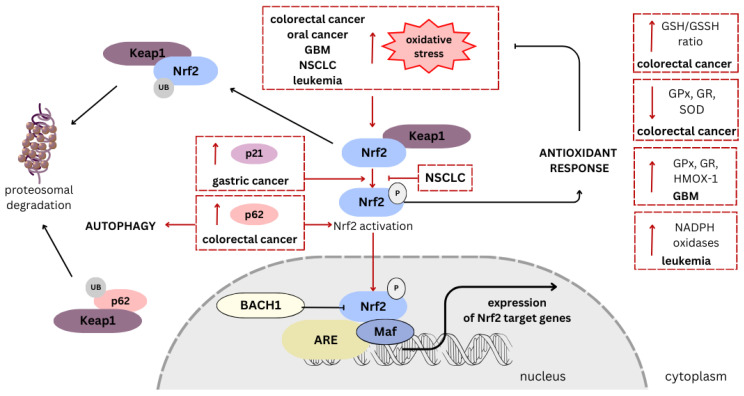
Mechanisms of Nrf2 pathway activation and signaling alterations induced by cannabidiol (CBD) in regard to various types of cancer. Oxidative stress is the main trigger for the antioxidant response through activation of the Nrf2 pathway. Firstly, it leads to the disruption of the Nrf2-Keap1 complex. The Nrf2 detachment facilitates its translocation to the nucleus, where it dimerizes with Maf proteins and binds to ARE, promoting gene transcription. Other proteins, such as p21 and p65, also contribute to enhancing Nrf2 activation. Conversely, under normal conditions, Nrf2 and p62 are ubiquitinated and degraded in the proteasome. In multiple types of cancer, CBD enhances ROS production, inducing oxidative stress [70,73,74,75,76,86,87]. After exposure of colorectal cancer cells to CBD, an increase in the p65 level was observed [71], while in gastric cells, an increase in the p21 level was demonstrated [88]. In NSCLC cells, CBD causes massive oxidative stress while at the same time reducing Nrf2 activation [73,86]. CBD treatment modulates enzyme and protein levels associated with the Nrf2 pathway and antioxidant response. An increase in GSH/GSSH ratio and a decrease in GPx, GR, and SOD levels were observed in colorectal cancer cells [70]. In turn, in GBM the level of GPx, GR, and HMOX-1 were elevated [75,76], while in leukemia, the level of NADPH oxidases increased [74]. An arrow-ended line indicates stimulation, while a dash-ended line represents inhibition; P denotes phosphorylation. Frames and lines in red indicate CBD’s impact on Nrf2 pathway in specified cancer’s type. NRF2, nuclear factor erythroid 2-related factor 2; Keap1, Kelch-like ECH-associated protein 1; BACH1, BTB domain and CNC homolog 1; Maf, musculoaponeurotic fibrosarcoma proteins; ARE, antioxidant response element; GBM, glioblastoma multiforme; NSCLC, non-small-cell lung cancer; GPx, glutathione peroxidase; GR, glutathione reductase; SOD, superoxide dismutase; HMOX-1, heme oxygenase-1; GSH/GSSH, reduced/oxidized glutathione.

The literature data have suggested that CBD can activate the Nrf2 pathway, increasing the expression of antioxidant and detoxification enzymes (Figure 3). This activation occurs through the binding of CBD to cannabinoid receptors, particularly CB1 and CB2 receptors, as well as non-cannabinoid receptor pathways. One of the mechanisms of the CBD-mediated regulation of the Nrf2 pathway is the increase in the level of Nrf2 pathway activators, such as p21 and p62, and the reduction in the level of its inhibitors, including cytosolic ECH-like proteins associated with Kelch1, Keap1, and nuclear Bach1 [85]. Such observations were noted in a model of skin keratinocytes. Interestingly, Casares et al. demonstrated that in keratinocytes, CBD is a weak Nrf2 activator but a good BACH1 inhibitor [83]. In this study, CBD selectively stimulated the expression of a limited subset of Nrf2-induced target genes, such as *HMOX-1* and *p62*, but was dramatically less potent in inducing the expression of other Nrf2 target genes, such as aldo-ketoreductases [83]. Other phytocannabinoids, such as CBC and CBG, were found to be less potent in inducing HMOX-1, and their acidic forms were inactive [83].

On the contrary, the proteomic data obtained from the skin keratinocytes of nude rats treated topically with 4 μM CBD after UVA/B irradiation (in vivo) indicate a significant decrease in the UV-induced levels of Nrf2 and Cu, Zn-superoxide dismutase (Cu, Zn-SOD) [84]. Interestingly, dose-dependent effects of CBD on the Nrf2 pathway were also observed. In a study by Böckmann et al., up to 6 μM CBD induced upregulation of Nrf2 and HMOX-1 expression, while this phytocannabinoid in 10 μM concentration downregulated Nrf2 and promoted autophagy in human umbilical vein endothelial cells [82]. 

The exact mechanisms through which CBD triggers Nrf2 activation are not fully elucidated, but it is believed that the antioxidant and anti-inflammatory properties associated with Nrf2 activation could be beneficial in combating oxidative stress-related diseases and certain types of cancer.

### 5.1. Colorectal Cancer

Chronic intestinal inflammation bears the risk of carcinogenesis, indicating the role of sphingolipids, which mediate cellular functions, specifically migration, proliferation, and apoptosis [89]. A recent study demonstrated that prolonged colitis in patients with inflammatory bowel disease (IBD) may lead to the development of colitis-associated colorectal cancer [90]. These findings represent the theory that a compound with anti-inflammatory properties could be beneficial in reducing inflammation associated with colorectal cancer and potentially inhibiting cancer cell growth.

Hence, in the inflamed colon treated with CBD, pro-inflammatory intracellular pathways were found to be downregulated, thereby preventing the production of cytokines [91]. Another study demonstrated that CBD administration in mice with colon cancer caused a significant decrease in the levels of IL-6 and IL-8 [92]. Research papers suggest that CBD might induce apoptosis and cell cycle arrest in colorectal cancer, preventing their uncontrolled proliferation. In the study of Cerretani et al., it has been shown that decreased survival of HT-29 cells and apoptosis induction after CBD treatment is CB receptor-independent, and it has been suggested that CBD-triggered rapid increase of ROS is mainly responsible for enhanced oxidative stress [70]. In this way, to protect cells from lethal toxicity, the main antioxidant Nrf2-Keap1 pathway is activated. Keap1 degrades, resulting in Nrf2 nucleus translocation [71]. Wang et al. noted that in p53 wild-type colorectal cancer cells, these processes are associated with p62 overexpression and macroautophagy activation and lead to unfavorable anti-apoptotic effects, which might be reversed by inhibiting autophagy [71]. On the other hand, Cerretani et al. observed a significant reduction in the GSH/GSSG ratio in CBD-treated cells, confirming the presence of oxidative stress, as well as decreased activity of antioxidant enzymes, Nrf2 target proteins, GR, GPx, and catalase (CAT) [70]. The authors suggest that the increased demand for reducing equivalents necessary to maintain GSH levels results from excess ROS causing oxidative stress. Additionally, the activation of an antioxidant response on ROS generation might depend on the cellular defense system and the phenotype of complex set of proteins associated with apoptosis and autophagy. Conversely, in the same study, the effects of the treatment with THC and the synthetic cannabinoid CB83 were significantly different from CBD, suggesting the involvement of CB receptors and not triggering oxidative stress [70]. Nevertheless, some changes in the antioxidant enzyme activity were noted, with GR and GPx showing increased activity and reduced CAT activity. Interestingly, an in vivo study showed that mice receiving a treatment of 5 mg/kg CBD exhibited a significant increase in SOD, GPx, and GR activities, which determine beneficial anti-angiogenesis and anti-metastatic effects [92].

Studies have indicated that THC might inhibit the proliferation of colorectal cancer cells, slowing down tumor growth and progression. In this regard, enhanced death of colon cancer cells was elucidated by THC-triggered activation of caspase-3 and RAS-MAPK/ERK, as well as PI3K-AKT signaling, with BAD suppression mediated by CB1R stimulation [93]. 

Moreover, Aviello et al. investigated the possible chemopreventive effect of CBD in the model of colon cancer induced by azoxymethane (AOM) in mice [94]. The researchers found that CBD protected DNA from oxidative damage, increased endocannabinoid levels, and reduced cell proliferation in a CB1-, TRPV1-, and PPARγ-antagonist-sensitive manner [94].

It is worth noting that cannabinoids’ potential to alleviate pain and other symptoms associated with colorectal cancer and its treatments could improve the quality of life for patients undergoing conventional therapies [95]. Furthermore, it has been observed that in vivo treatment with Δ9-THC targeting CB2R in immune CD8^+^ T cells significantly mitigated cancer-associated cachexia and muscle atrophy [96].

### 5.2. Oral Cancer

In the study of Loubaki et al., the treatment of oral cancer cells with a low concentration of a mixture of cannabinoids (CM-8 component solution obtained from Sigma-Aldrich, St. Louis, MO, USA Cat. Number = C-219-1ML) induced ROS-based autophagy and oxidative stress [87]. In addition to ROS, intracellular GSH, which plays a pivotal role in retaining homeostasis, increased following exposure to CM [87]. Additionally, downregulation of cancer-related pathways such as NF-kB was shown after cannabinoids exposure [87]. The authors showed that the mixture of cannabinoids, particularly at a dose of 1 µg/mL, inhibited oral cancer cell proliferation through diverse mechanisms, including apoptosis and autophagy. 

Moreover, it is essential to note that, according to Li et al., CBD might alleviate the severity of chemotherapy-induced oral mucositis [81]. In the in vitro model of human oral keratinocytes treated with 5-fluorouracil (5-FU), CBD attenuated ROS overproduction, upregulated the expression levels of Nrf2 and the antioxidant enzymes HMOX-1 and NQO1, and decreased the level of Keap1. Further, mucosal inflammation has been reduced in vivo, improving the clinical scores and systemic conditions of the 5-FU-treated mice exposed to CBD. Increased SOD1, HMOX-1, and NQO1 levels in tongue tissues confirmed CBD treatment’s anti-inflammatory and antioxidant effects [81].

### 5.3. Gastric Cancer

CBD’s anti-inflammatory properties are also of interest in the context of gastric cancer. Chronic inflammation is a known risk factor for cancer development, and CBD’s ability to mitigate inflammation could contribute to its potential as an adjuvant therapy for gastric cancer. It has been shown that in a rat model, *Cannabis* extract increased the antioxidant mechanisms in terms of drug-induced oxidative stress (stimulation of gastric acid secretion with carbachol, pentagstrin, or histamine) [97]. Pre-treatment with *Cannabis*, administered subcutaneously in doses of 5, 10, and 20 mg/kg, increased GSH content compared to the control group, leading to decreased gastric mucosal damage [97]. It has also been suggested that the protective role of *Cannabis* sativa extract might be elucidated by its ability to reduce inflammatory cytokine production [97].

The study by Zhang et al. demonstrated that CBD significantly induced cell cycle arrest at the G0–G1 phases and inhibited the proliferation and colony formation of SGC-7901 cells [72]. Moreover, they confirmed a CBD-induced increase in intracellular ROS levels. Interfering with the cell cycle might be associated with p21 upregulation and p53 downregulation, whereas the interaction between p21 and Nrf2 stabilizes Nrf2, leading to an increased response to oxidative stress [88,98]. In addition, CBD significantly increased Bax expression levels, decreased Bcl-2 expression levels and mitochondrial membrane potential, and then upregulated cleaved caspase-3 and cleaved caspase-9 levels, thereby inducing apoptosis in SGC-7901 cells [72]. 

In conclusion, cannabinoids, particularly CBD, are promising modulators of the Nrf2 pathway in the context of gastric cancer. Their potential to activate antioxidant responses, mitigate inflammation, and influence cancer cell survival mechanisms suggests a potential role in improving the therapeutic landscape for gastric cancer patients. However, further research, including well-designed clinical trials, is essential to fully comprehending the therapeutic potential of cannabinoids and their mechanisms of action in gastric cancer treatment.

### 5.4. Non-Small-Cell Lung Cancer (NSCLC)

The anti-inflammatory properties of CBD are also of interest in the context of NSCLC. Chronic inflammation is known to play a role in the development and progression of lung cancer. CBD’s ability to modulate inflammatory responses could contribute to its potential as an adjunct therapy for NSCLC. CBD-induced massive oxidative stress was noted in the lung cancer adherent cells after 24 h incubation at a 10 μM dose [86]. In this study, differences in the intensity of ROS generation and expression of antioxidant genes after CBD exposure have been observed in a cell-dependent manner [86]. Interestingly, the authors indicated that CBD exhibits both pro- and antioxidant functions. Another study has shown that in chemotherapy-resistant NSCLC cells with significantly higher endogenous Nrf2 expression, CBD can reduce Nrf2 pathway activation [73]. By doing so, CBD could induce oxidative stress in the tumor microenvironment via enhanced intracellular ROS generation. Additionally, CBD has been suggested to promote apoptosis in NSCLC cells through TRPV2 activation and an increase in intracellular Ca^2+^ levels, contributing to the suppression of tumor growth [73]. Pro-apoptotic effects of CBD treatment were observed by a higher expression of the apoptosis markers, cleaved caspases 3 and 9, compared to cisplatin treatment [73].

### 5.5. Leukemia

Khodakarami et al. reviewed the potential role of Nrf2 in leukemia, emphasizing the dualistic nature of this transcription factor [99]. The cytoprotective role of activated Nrf2 can be profitable in maintaining redox homeostasis and inflammatory responses in non-malignant cells. However, in cancer cells, Nrf2 overexpression could contribute to escaping from apoptosis and developing resistance to therapy. Therefore, downregulating or silencing Nrf2 seems to be a more relevant approach against leukemia [99].

In response to CBD exposure, McKallip et al. observed a significant increase in ROS production in treated Jurkat cells, as well as upregulation of NAD(P)H oxidases Nox4 and p22phox, the enzymes playing an important role in ROS level regulation and oxidation balance [74]. Treatment with CBD in ≥2.5 μM doses resulted in limited cell survival, indicating the high sensitivity of leukemia cells to the pro-apoptotic CBD’s effect. However, the explanation of this phenomenon is not fully elucidated. The researchers showed that after inhibition of CB2R, the level of NAD(P)H oxidases was not significantly changed, suggesting that CBD’s anti-tumor activity can be elucidated by agonistic receptor interaction in leukemia cells. Additionally, alterations in CB2-dependent p38 signaling have been demonstrated in the study [74].

Still, CBD’s anti-inflammatory properties could contribute to its potential therapeutic effects in leukemia treatment. Therefore, the properties of cannabinoids have been demonstrated in the study concerning polymorphonuclear leukocytes (PMN), in which *Cannabis* extract containing 5% CBD and <0.2% THC showed the ability to inhibit the oxidative metabolism and production of the pro-inflammatory cytokine TNF-α [61].

### 5.6. Glioblastoma

Research suggests that both ∆9-THC and CBD can impact the Nrf2 pathway in glioblastoma cells. Studies have shown that CBD can activate Nrf2, increasing antioxidant and detoxification enzyme expression. This activation may help protect cells from oxidative stress and inflammation, which are key contributors to cancer progression. Additionally, CBD has been found to induce apoptosis (programmed cell death) in glioblastoma cells, potentially inhibiting their growth [21,100].

In the study conducted by Massi et al., excessive ROS production in U87 glioma cells due to 25 μM CBD exposure has been demonstrated [75]. Meanwhile, the authors examined the level of intracellular GSH, showing significant GSH depletion in cells exposed to CBD for 6 h at the same concentration. However, the activity of GSH-associated enzymes (GPx and GR) was significantly stimulated by exposure to either 10 μM or 25 μM CBD [75]. Based on these results, they proposed the mechanism by which CBD induces apoptosis in human glioma cells, involving the induction of oxidative stress, followed by the activation of the caspase cascade. Similarly, in another study, CBD induced a robust increase in ROS in glioma stem cells [76]. In this regard, cell viability decreased, and the antioxidant agent, vitamin E, could reverse the effect. In turn, in an in vivo model, the antioxidant response was demonstrated via Nrf2 activation and upregulation of SLC7A11 (xCT) and HMOX-1, suggesting the disadvantageous mechanism underlying resistance to the CBD-based redox therapeutic [76]. 

Extensive research by Juknat et al. concerning the anti-inflammatory effects of cannabinoids on BV-2 microglia cells confirmed the upregulation of Nrf2 target genes as a result of pro-inflammatory stimulation [77,78,79]. The authors took into consideration both natural (CBD, ∆9-THC) and synthetic compounds (dimethyl heptyl-cannabidiol), obtaining similar results that indicate downregulation of pro-inflammatory genes (Il1β, Il6, and TNF) as well as upregulation of genes related to oxidative stress (*Trb3, Slc7a11/xCT, HMOX-1, Atf4, Chop,* and *p8*) [77]. Additionally, anti-inflammatory events were observed after CBD and ∆9-THC treatment due to the reduction in NF-κB pathway activation and, only for CBD, the activation of STAT3 [78]. Thus, the utility of cannabinoids could be considered regarding the prevention of neuropathology and cancer development.

## 6. Targeting the Nrf2 Pathways by Cannabidiol and Its Combination with Other Compounds

Several studies confirm that the anti-inflammatory and anti-cancer effects of cannabinoids can be enhanced when combined with other components compared to treatment with isolated active compounds alone (Table 2). Specifically, co-treatment has the potential to alleviate side effects and improve therapy effectiveness. Moreover, understanding the “entourage effect” caused by the complexity of the various *Cannabis* compounds, including ∆9-THC, CBD, and other cannabinoids, is crucial. This effect refers to the potential synergy between different compounds in the plant, which might enhance therapeutic outcomes [101]. Thus, examples of reduced tumor growth resulting from co-treatment with *Cannabis* were observed in various cancer models, including glioblastoma [102,103], breast cancer [104], or melanoma [105]. However, the relationship between inhibiting tumor progression and Nrf2 modulation after *Cannabis* exposure is still not fully elucidated.

In the motoneuron-like cell line NSC-34 model, the anti-inflammatory and antioxidant effects of CBD and CBG, individually and in combination, were investigated in neuroinflammation [106]. Mammana et al. observed that the co-administration of higher doses of CBG with CBD (5 μM + 5 μM) is more effective in inhibiting NF-κB nuclear translocation and increasing cytoplasmic IκBα levels as compared to lower doses, namely 2.5 μM + 2.5 μM, respectively. Also, the study confirmed the impact of CBD on cellular redox status by showing an increase in Nrf2 nuclear translocation, whereas CBG alone did not activate Nrf2. Nonetheless, CBD + CBG co-treatment holds promise as an approach to addressing neuroinflammation [106]. The combination of the two natural products with antioxidant activity, CBD and isothiocyanate-moringin, was studied in 2016 to assess their anti-inflammatory potential [107]. When LPS-stimulated murine macrophage RAW cells were treated with the compounds either individually or in combination, a more significant increase in Nrf2 activation was observed in cells treated with moringin alone and in combination with CBD than in cells treated with CBD alone. This suggests that exposure to CBD and moringin together enhances the response to oxidative stress and could be considered an anti-inflammatory treatment [107]. 

The anti-cancer properties of synthetic CBD and THCV in combination with doxorubicin were investigated as an approach for chemo-resistant triple-negative breast cancer (TNBC) by Kalvala et al. [108]. The authors suggested a chemo-sensitizing effect of the combination and the ability to overcome the resistance based on Western blot and proteomic analysis in MDA-MB-231 xenografts in athymic nude mice. This analysis demonstrated the downregulation of Nrf2 and other targets such as HMOX-1, SOD, Bcl-xL, P-38 MAPK, TGF-β, PD-L1, CD133, NF-kB, CAT, tenascin, SP1, and the NLRP3 inflammasome, which were observed to be upregulated in tumors in response to standard therapy [108]. These alterations may be associated with a downregulation of H3K4 histone methylation as well as H2K5 histone acetylation, which has also been reported in the study. The linkage between these outcomes was suggested as an explanation for the beneficial input of CBD and THCV into overcoming DOX resistance in TNBC [108]. Interestingly, improved outcomes were obtained in the Wang et al. study mentioned earlier, which combined CBD with the heat shock protein 70 (Hsp70) inhibitor, PES-CI [71]. Co-treatment of colorectal cells with PES-CI and CBD resulted in enhanced apoptosis via overproduction of cleaved caspases-9/3. However, the critical profitable outcome was the ability of PES-CI to reverse macroautophagy activated as a protective mechanism against CBD-induced ROS overproduction [71].

**Table 2:** The overview of CBD and its combination with other compounds regarding their impact on Nrf2 pathway.

Combination	Condition, Experimental Model	Key Findings of Nrf2 Modulation	References
CBD and tetrahydrocannabivarin (THCV) with doxorubicin (DOX)	**Breast cancer** Triple-negative breast cancer MDA-MB-231 DOX resistant and wild-type control cells	CBD and THCV downregulated CAT, SP1, NLRP1, SOD2 genes. CBD + DOX and THCV + DOX combo treatment reduced CAT, HMOX-1, SP1, NLRP3 levels.	[108]
CBD with moringin	**Inflammation** Murine macrophage cells RAW 264.7	The combination of CBD-moringin enhanced Nrf2 level more than CBD alone.	[107]
CBD with CBG	**Neuroinflammation** Motoneuron-like cells NSC-34 treated with medium of LPS-stimulated RAW 264.7 macrophages	Co-administered CBD and CBG increased Nrf2 translocation.	[106]
CBD with PES-CI	**Colorectal cancer** Human adenocarcinoma colon cells HCT116 (p53 wild type) HCT116 (p53 double knockout), SW480, LS174 (p53wild type) SCID mice xenograft model (injected with HCT116 p53 wild-type or p53 double knockout cells)	Hsp70 inhibitor potentiates the anti-tumor effect of CBD with the decrease in ROS and corresponding decrease in the Keap1 expression results in the nuclear translocation of Nrf2	[71]

## 7. Conclusions

The bioactive compounds of *Cannabis* sativa have always attracted scientific interest due to their broad effects spectrum, including anti-cancer properties. Since Nrf2 holds a central role in cytoprotection, targeting the activation of this pathway may positively impact inflamed cells, diminishing pro-inflammatory processes and decreasing the risk of cancer development. Simultaneously, excessive Nrf2 nuclear translocation has been associated with chemo- and radio-resistance. Thus, exploring the contribution of *Cannabis* to Nrf2 regulatory processes provides new insights into both the potential benefits and limitations of these compounds, concerning further preclinical and clinical studies. In this review, we demonstrated key findings on how CBD and other cannabinoids suppress oncogenic effects by involving their pro- and antioxidant properties, both directly and indirectly associated with Nrf2 signaling. The above findings prompt that the *Cannabis*-induced Nrf2 modulation cannot be interpreted explicitly due to the complexity of the tumorigenesis molecular background. Nevertheless, the combined treatment with other compounds may leverage the anti-tumor properties of cannabinoids while concurrently diminishing the protective mechanisms of malignant cells against anti-cancer actions. In conclusion, as cannabinoids and their derivatives show more and more therapeutic importance, their redox modulatory activity needs to be fully elucidated regarding their effectiveness against cancer. Future studies should also consider the crosstalk between Nrf2’s anti-inflammatory potential in normal cells and the tumor microenvironment with an inflammatory profile by interfering with scavenging free radicals, reducing metal ions, and protecting oxidation processes.

## Figures and Tables

**Table 1:** The summary of the key findings of Nrf2 modulation regarding cancer and inflammation-related conditions.

Condition	Compound	Experimental Model	Key Findings of Nrf2 Modulation	References
Colorectal cancer	∆9-THC (natural) CBD (natural) CB83 (synthetic)	Human colorectal carcinoma cells HT-29	Significantly reduced glutathione/ oxidized glutathione ratio in CBD-treated cells and significantly increased in CB83-treated cells. CBD, ∆9-THC, and CB83 reduced catalase activity. The activities of glutathione reductase and glutathione peroxidase were significantly increased in cells exposed to ∆9-THC and significantly decreased in those treated with CBD.	[70]
	CBD (natural)	Human adenocarcinoma colon cells: HCT116 (p53 wild type) HCT116 (p53 double knockout), SW480, LS174 (p53wild-type) SCID mice xenograft model (injected with HCT116 p53 wild-type or p53 double knockout cells)	CBD treatment induces ROS production and stimulation of the Keap1-Nrf2 antioxidant pathway in p53 wild-type cells.	[71]
Gastric cancer	CBD (natural)	Human gastric cancer cells SGC-7901	CBD markedly enhanced ROS intracellular levels and increased p21 level.	[72]
Non-small-cell lung cancer (NSCLC)	CBD (natural)	Large cell carcinoma cells H460 (cisplatin-resistant) Adenocarcinoma cells A549 (cisplatin-resistant) NSC mice xenograft model (injected with H460 cells)	CBD treatment decreased Nrf2 expression in cisplatin-resistant NSCLC cells. Reduction in tumor progression and metastasis through inhibition of cell growth by reducing Nrf2 expression, increasing ROS generation, and targeting TRPV2.	[73]
Leukemia	CBD (natural)	Murine lymphoma cells EL-4 Human leukemia cells Jurkat and MOLT-4 C57BL/6 mice model (injected with EL-4 cells)	CBD increased production of ROS as well as upregulated the NAD(P)H oxidases -Nox4 and p22phox.	[74]
Glioblastoma	CBD (natural)	Human glioma cells U87	CBD induced production of ROS, depletion of intracellular glutathione and increased activity of glutathione reductase and glutathione peroxidase enzymes.	[75]
		Human glioma cells U251 Tissue-derived glioma stem cells (GSC lines 387 and 3832) Athymic nu/nu mice model (injected with GSC lines 3832 or 387)	CBD induced nuclear translocation and activation of Nrf2. Inhibited expression of Sox2 but upregulated expression levels of SLC7A11 (xCT) and HMOX-1.	[76]
Neuroinflammation (microglia)	CBD (natural) ∆9-THC (natural) Dimethylheptyl-cannabidiol (DMH-CBD) (synthetic)	Immortalized murine microglial cells BV-2 stimulated with lipopolysaccharide (LPS)	CBD induced HMOX-1, Slc7a11 (xCT) and Bach1 upregulation. CBD and less THC treatment caused Herpud, Gclm, Gstm6, HMOX-1, NQO1 and Gstm1 upregulation. In cells treated with DMH-CBD the expression of Trb3, Slc7a11 (xCT), HMOX-1, Atf4, Chop, and p8 were upregulated.	[77,78,79,80]
Neuroinflammation (motor neurons)	CBG (natural)	Motor neurons NSC-34 treated with medium of LPS-stimulated RAW 264.7 macrophages	CBG pre-treatment reduced SOD1 levels and restored Nrf2 levels in cells treated with medium of LPS-stimulated macrophages.	[66]
Chemoprevention	CBD (natural) Hexocannabitriol (synthetic)	Human epidermal keratinocyte-ARE-luciferase cells (HaCaT-ARE-Luc)	Hexocannabitriol showed a very potent Nrf2 activation, greater than CBD-treated keratinocytes.	[6]
Oral mucositis	CBD (natural)	Human oral keratinocytes from 5-fluorouracil-induced oral mucositis; C57BL/6N mice model (treated with 5-fluorouracil)	CBD caused increasing expression and nuclear translocation of Nrf2 and decreasing Keap1. Upregulated the expression levels of HMOX-1and NAD(P)H quinine oxidoreductase 1 (NQO1).	[81]
Atherosclerosis	CBD (natural)	Human Umbilical Vein Endothelial Cells (HUVEC)	CBD showed a concentration-dependent increase of Nrf2 as well as HMOX-1 mRNA and protein level.	[82]
Skin inflammation	CBD (natural)	Normal human epidermal keratinocytes (NHEK); HaCaT-ARE-Luc cells	CBD dramatically reduced BACH1 total and nuclear levels and enhanced HMOX-1 and p62 gene expression.	[83]
	CBD (natural)	RH-FOXN1RNU rats irradiated with UVA/B	CBD reduced the dramatic Nrf2 increase and NADPH-dependent diflavin oxido reductase 1 (D4ABT4) and SOD after UVA/UVB exposure	[84]
Diabetic cardiomyopathy	CBD (natural)	C57/BL6J mice model (treated with streptozotocin)	CBD reduced the increased activity of NADPH oxidases, SOD and reversed GSH/GSSG ratio.	[57]
